# Optimizing the performance of photocatalytic H_2_ generation for ZnNb_2_O_6_ synthesized by a two-step hydrothermal method†

**DOI:** 10.1039/c8ra01624k

**Published:** 2018-04-13

**Authors:** Yutong Chun, Mufei Yue, Pengfei Jiang, Shijian Chen, Wenliang Gao, Rihong Cong, Tao Yang

**Affiliations:** College of Chemistry and Chemical Engineering, Chongqing University Chongqing 401331 P. R. China pengfeijiang@cqu.edu.cn taoyang@cqu.edu.cn; College of Physics, Chongqing University Chongqing 401331 P. R. China

## Abstract

Semiconductor-based photocatalytic H_2_ generation is a promising technique and the development of efficient photocatalysts has attracted great attention. Columbite-ZnNb_2_O_6_ is a wide-bandgap semiconductor capable of photocatalytic water splitting. Here we employed a two-step hydrothermal method to first dissolve Nb_2_O_5_ with a highly basic aqueous solution and further react it with Zn^2+^ to form nanosized ZnNb_2_O_6_. The reaction time plays an important role on its morphology and photocatalytic performance in water reduction. The sample synthesized through 7 days of reaction was the optimal one with an appropriate crystallinity and a large specific surface area, however the severe surficial defects prohibited its photocatalytic activity in pure water. The H_2_ generation at a rate of 23.6(5) μmol h^−1^ g^−1^ emerged when 20 vol% methanol was used as the hole-sacrificial agent. Most remarkably, once metal or metal oxide cocatalysts, including Pt, Au, NiO, RuO_2_, Ag_2_O, and Pd/PdO, were loaded appropriately, the photocatalytic H_2_ generation rate ultimately achieved 3200(100) or 680(20) μmol h^−1^ g^−1^ with or without using methanol, respectively. Apparent quantum yields (AQYs) at 295 nm were investigated by changing the experimental parameters, and the optimal AQYs are 4.54% and 9.25% in water and methanol solution, respectively. Further post-modifications like bandgap engineering may be performed on this highly efficient nano-ZnNb_2_O_6_.

## Abstract 2

Nanosized ZnNb_2_O_6_ synthesized hydrothermally is highly efficient and stable in photocatalytic water reduction with an optimal AQY of 9.25%.

## Graphical abstract

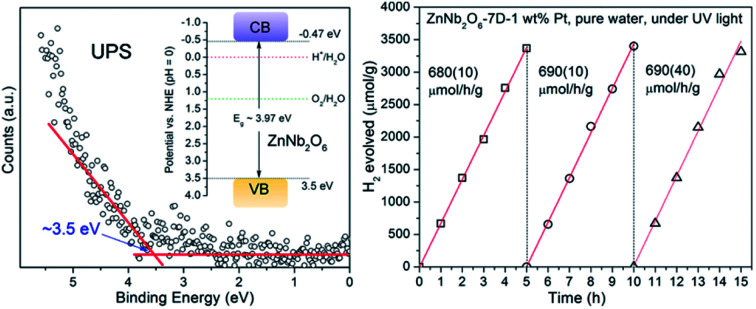

## Introduction

The technology of semiconductor-based photocatalytic water reduction using solar energy has been considered as one possible solution to the incoming energy crisis. Research on developing new semiconductor photocatalysts is always active.^1–3^ Due to the d^10^ electron configuration of Nb^5+^, people have developed several niobium-containing oxide photocatalysts, for example, K_4_Nb_6_O_17_,^4,5^ Ag_2_Nb_4_O_11_,^6^ Ba_5_Nb_4_O_15_,^7^ Bi_3_NbO_7_,^8^ and ZnNb_2_O_6_.^9–11^

We are interested in columbite-type ZnNb_2_O_6_, which was already suggested to be potentially applicable as dielectric ceramics or luminescent materials.^12–15^ The columbite structure of ZnNb_2_O_6_ is constructed by a distorted hexagonal-closed-packed ionic sub-lattice along the *a*-axis, where half of the octahedral cavities are occupied by either Zn^2+^ or Nb^2+^ in an ordered mode as shown in Fig. S1 in the ESI.† With regards to its photocatalytic activity in water splitting, A. Kudo reported in 1999 that ZnNb_2_O_6_ loaded with 0.5 wt% NiO possessed a high activity (54 and 21 μmol h^−1^ g^−1^ for H_2_ and O_2_ production, respectively) under the irradiation of 450 W high pressure mercury lamp.^9^ ZnNb_2_O_6_ powder catalyst in that study was prepared by typical high temperature solid state reaction, which must be composed of poly-crystallites with the size in the micrometer level.

In 2009, J. S. Lee performed the band structure engineering on ZnNb_2_O_6_ by doping V^5+^ at the Nb^5+^ site, in order to acquire the visible light response.^16^ Indeed, the bandgap energy was reduced from 3.98 eV at *x* = 0 to 2.5 eV at *x* = 0.06, and the apparent quantum yields were 0.12% and 1.5% for H_2_ and O_2_ generation at 420 nm, respectively, in aqueous solutions containing sacrificial agents. Very recently, W. D. Shi selected ZnNb_2_O_6_ to combine with both N-doped graphene quantum dots and g-C_3_N_4_ to construct a composite catalyst with efficient heterojunctions, and eventually the visible light driven water reduction activity of g-C_3_N_4_ was significantly boosted.^17^

It is well known that the semiconductor-based photocatalytic reaction contains three successive processes after the photons been absorbed, including the formation of the photo-excited electrons and holes, their migration to the particle surface, and the surficial reaction with, for example, water molecules. Particularly, ZnNb_2_O_6_ is a relatively resistive semiconductor, and the migration of electrons/holes to the surface is very difficult due to the high resistivity, as a consequence, only the photo-excited electrons and holes formed spatially close to the surface can contribute to the catalytic reactions, while most electrons and holes in the inner part of the bulk crystallites will annihilate inevitably. The strategy to solve this problem is simply preparing nanosized catalysts, which would not only greatly decrease the distance of the migration length to the surface, but also offer a higher specific surface area along with more active sites for catalytic reactions.

Herein this work, Nb_2_O_5_ was dissolved in the highly basic solution (KOH aqueous solution) in a closed system, and further reacted with Zn^2+^ to form phase-pure ZnNb_2_O_6_ nanoparticles at hydrothermal conditions. Its band structure was explored by density functional theory calculations, and experimentally, the potentials of valence band maximum and conduction band bottom were estimated by the ultraviolet photoelectron spectroscopy and UV-Vis diffuse reflectance spectra to be 3.5 and −0.47 eV, respectively. The nanosized ZnNb_2_O_6_ synthesized hydrothermally exhibited a high specific surface area, and with the assistance of the sacrificial agents, it possessed an intrinsic photocatalytic activity in H_2_ production under UV light irradiation, however, no activity was detected in pure water, indicating the surficial defects may cause the high recombination rate of electrons and holes. Then, various metal or metal oxide cocatalysts (including Pt, Au, RuO_2_, NiO, Pd/PdO, and Ag_2_O, identified by X-ray photoelectron spectroscopy) were deposited on the nanoparticle surface, and the photocatalytic H_2_ production rate increased significantly up to 680(20) and 3200(100) μmol h^−1^ g^−1^ in pure water and methanol aqueous solution, respectively. The high stability of ZnNb_2_O_6_ was confirmed by cycling experiments and the powder X-ray diffraction after the photocatalysis, in addition, the optimal apparent quantum yields (AQY) under irradiation of the monochromatic light at 295 nm were 4.54% in pure water and 9.25% in 20 vol% methanol aqueous solution, respectively. Our efforts on optimizing the intrinsic photocatalytic performance of ZnNb_2_O_6_ offers a new platform for further post-modifications, like bandgap engineering or constructing a composite catalyst system.

## Experimental

### Synthesis of catalyst and loading of cocatalysts

A two-step hydrothermal method was applied to synthesize ZnNb_2_O_6_ photocatalyst.^11^ First, a pre-reaction was performed to dissolve Nb_2_O_5_ (0.10 g) using 7.5 mL of KOH aqueous solution (4 mol L^−1^) in a closed system at 180 °C for 10 hours. Then a pre-calculated amount of Zn(Ac)_2_·2H_2_O with a molar ratio of Zn : Nb = 1 : 2 was loaded into this solution. Afterwards, pH was adjusted to ∼6 using concentrated hydrochloric acid under stirring. Finally, a turbid solution will be obtained and further loaded into a 25 mL Teflon-lined autoclave, sealed and kept at 240 °C for 3, 5, 7, and 9 days, respectively. After the reaction, the autoclave was naturally cooled to room temperature. The so-obtained solid product was washed extensively for several times with alcohol and water, and then dried at 60 °C. The resultant white powder sample was donated as ZnNb_2_O_6_-3D, -5D, -7D, -9D, respectively. The comparison sample of bulk-ZnNb_2_O_6_ was synthesized by typical high temperature solid state reaction. Stoichiometric reagents of ZnO and Nb_2_O_5_ were mixed and heated at 850 °C for 15 hours.

The loading of cocatalyst was proceed using the reduction method in KBH_4_ aqueous solution. Typically, 100 mg of ZnNb_2_O_6_ catalyst and a pre-calculated amount of metal salt solution (H_2_PtCl_6_·6H_2_O, PdCl_2_, HAuCl_4_·4H_2_O, AgNO_3_, Ni(NO_3_)_2_·6H_2_O, or RuCl_3_) was mixed in 50 mL of distilled water. This mixed aqueous solution in a 100 mL beaker was ultrasonicated for 20 minutes. An appropriate amount of diluted KBH_4_ aqueous solution was added into the beaker very slowly. Finally, the obtained powder sample was washed extensively with water and dried at 60 °C for further usage.

### Characterizations

Powder X-ray diffraction (XRD) data were collected on a PANalytical X'pert diffractometer equipped with a PIXcel 1D detector with Cu Kα radiation. The operation voltage and current are 40 KV and 40 mA, respectively. Scanning electron microscopy (SEM) images were taken using a JSM-7800F electron microscope at the voltage of 3 kV and the working distance of 4 mm. The nitrogen adsorption–desorption experiments were performed at 77 K using a Quantachrome Quadrasorb SI analyzer. Prior to measurements, the samples were degassed at 300 °C for 10 hours, and the specific surface area was estimated according to the Brunauer–Emmett–Teller (BET) method. The UV-Vis diffused reflectance spectra (DRS) were recorded at room temperature using a UV-Visible-Near Infrared spectrometer (Shimadzu UV-3600) equipped with an integrating sphere attachment. BaSO_4_ was used as reflectance standard. The X-ray photoelectron spectra (XPS) and ultraviolet photoelectron spectroscopy (UPS) were acquired with an Escalab 250xi photoelectron spectrometer with Ag Kα X-ray source and Ar Kα ultraviolet ray source, respectively. Photoelectrochemical (PEC) measurements were performed in a typical three-electrode electrochemical on a Zahner electrochemical workstation and PP211, in which the ZnNb_2_O_6_ film on FTO substrate, Pt wire, and Ag/AgCl electrode were used as the working, counter, and reference electrodes, respectively. A 1.0 M Na_2_SO_4_ aqueous solution was used as the supporting electrolyte to maintain the stability of the film. The light irradiation was obtained from a monochromatic light (*λ* = 365 nm).

### Theoretical calculations

Theoretical studies were operated by the Vienna *ab initio* simulation package (VASP).^18^ The projector augmented-wave (PAW) method^19^ implemented in the VASP code was utilized to describe the interaction between the ionic cores and the valence electrons. The generalized gradient approximation (GGA) parameterized by Perdew, Burke, and Ernzerhof (PBE)^20^ was employed to describe the exchange-correlation potential in the standard Density Function Theory (DFT) calculations. For single point energy and density of states, a cutoff energy of 500 eV for the plane-wave basis and 7 × 7 × 7 Monkhorst–Pack G-centered *k*-point meshes were employed. The band structures *E*(*k*) were computed on a discrete *k* mesh along with high-symmetry directions.

### Photocatalytic activity evaluation

Photocatalytic H_2_ production was tested on a gas-closed circulation system equipped with a vacuum line (CEL-SPH2N system), a 150 mL Pyrex glass reactor with a quartz cover, and a gas sampling port that is directly connected to a gas chromatograph (Shanghai Techcomp-GC7900, TCD detector, molecular sieve 5A, N_2_ gas carrier). In a typical run, 50 mg of catalyst was dispersed by a magnetic stirring bar in 50 mL of pure water or 20 vol% methanol aqueous solution. A 5 °C recycling water bath was applied to keep the reaction vessel at a constant temperature. The irradiation source was generated by an external 500 W high-pressure mercury lamp laid on the top of the reaction vessel, whose emission spectrum is provided in Fig. S2, ESI.†

The apparent quantum yield (AQY) for H_2_ evolution under a monochromatic irradiation of 295 nm was calculated according to the following equation.
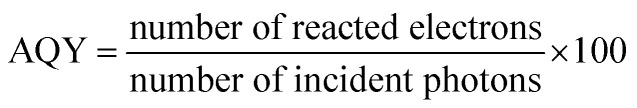


The number of reacted electrons is related to the H_2_ production rate, and the number of incident photons can be calculated according to the beam intensity measured by the Si-photodiode.

## Results and discussion

### Synthesis and morphology

The two-step hydrothermal method has the advantage in dissolving Nb^5+^ into the basic aqueous solution, and is beneficial for the preparation of nanosized crystallites in comparison with the traditional solid state reaction. The pH value, reaction temperature, and most importantly the reaction time strongly affect the purity and morphology of the final product. In literature, only the powder sample of ZnNb_2_O_6_ prepared at hydrothermal condition for 5 days was studied using the model reaction of photocatalytic degradation for methylene blue.^11^

**Fig. 1:**
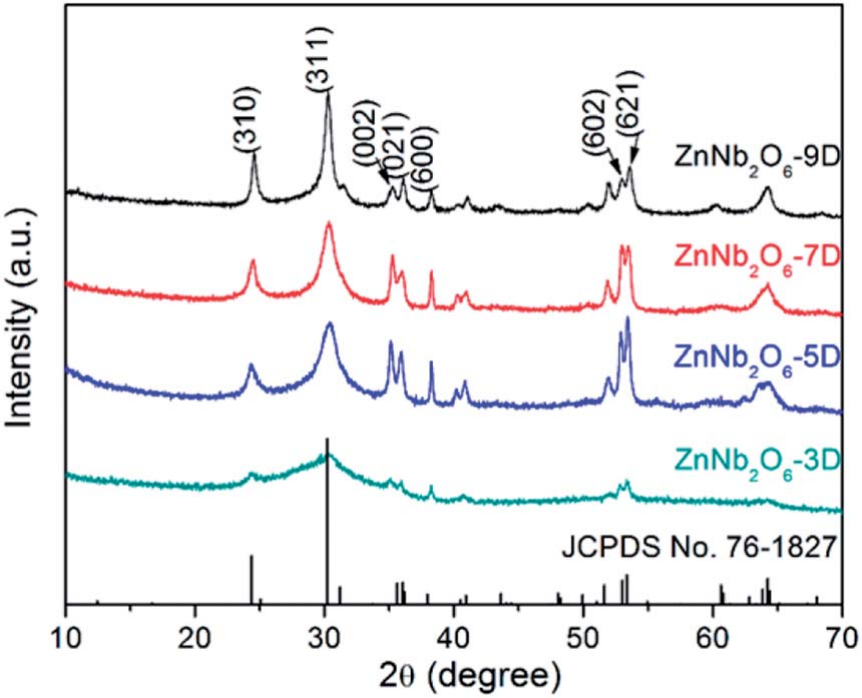
XRD patterns of ZnNb_2_O_6_ obtained by hydrothermal method for 3, 5, 7 and 9 days.

Herein this study, the powder products with the reaction times of 3, 5, 7, and 9 days were collected and characterized by XRD, SEM and BET analyses. As shown in Fig. 1, all the XRD patterns are consistent with the standard one of ZnNb_2_O_6_ (JCPDS No. 76-1827), indicating the phase purity. Some evolution tendency can be observed by comparing these four patterns. For instance, all the peaks in the sample ZnNb_2_O_6_-3D are broad. This is usually originated from the low crystallinity of the particles, which is reasonable due to the short reaction time of 3 days. For ZnNb_2_O_6_-5D, all the peaks become more obvious, but they can be classified into two groups. The first group comprises the broad peaks, as represented by two strong peaks with the indices of (310), (311), while the peaks with the indices of (002), (021), (600), (602) and (621) are relatively sharp, belonging to the second group. In a single-phase sample, the observable difference in peak shape is probably caused by the structural disordering and/or anisotropic crystal growth, and the latter one will result into low dimensional crystallites in morphology, *i.e.* needle- or petal-like. With further increasing the reaction time to 9 days, the difference in width between these two groups of peaks become smaller.

**Fig. 2:**
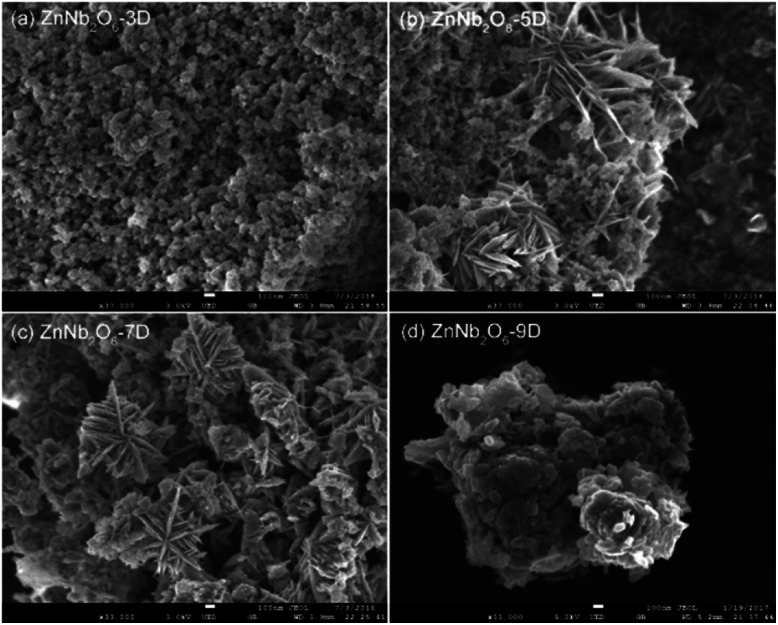
SEM images of (a) ZnNb_2_O_6_-3D, (b), ZnNb_2_O_6_-5D, (c) ZnNb_2_O_6_-7D and (d) ZnNb_2_O_6_-9D.

The evolution deduced from powder XRD patterns could be visualized directly by SEM images. As shown in Fig. 2, small nanoparticles with the size well below 100 nm aggregated when the reaction stopped after 3 days, and with the increasing of the reaction time, petal-like crystallites emerged and became the major component in ZnNb_2_O_6_-7D, however, the petal-like morphology generally disappeared in ZnNb_2_O_6_-9D due to the sufficient crystal growth. Accordingly, the specific surface area estimated using BET method exhibited a decreasing tendency from 101, 82, 61 to 41 m^2^ g^−1^ for 3D, 5D, 7D, and 9D samples, respectively (see Fig. S3, ESI†).

### Light harvesting ability and band structure analysis

**Fig. 3:**
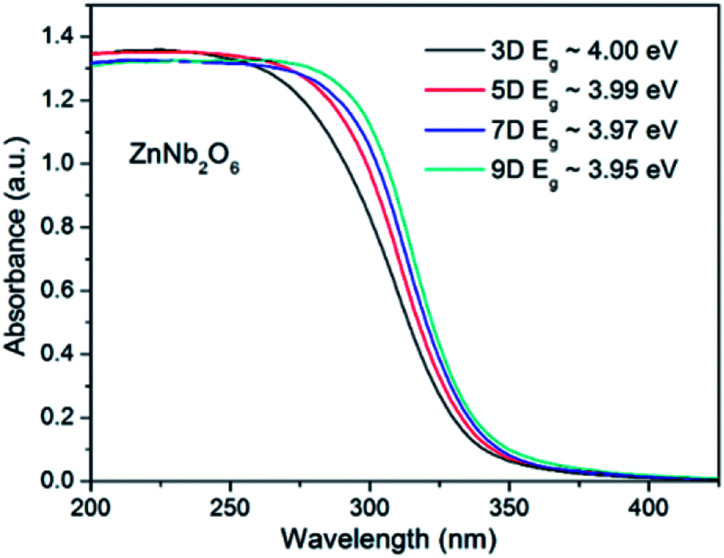
UV-Vis reflectance spectra of ZnNb_2_O_6_-3D, -5D, -7D, and -9D. The bandgap energies provided here were estimated from their differential curves.

Light harvesting is the first stage of photocatalysis, and the UV-Vis diffuse reflectance spectra for all four ZnNb_2_O_6_ samples were provided in Fig. 3. An obvious smooth and steep edge can be seen with only slight differences among these samples, which is an indication of the typical semiconducting-type bandgap transition. The bandgap energy, estimated by the negative peak at the differentiate curves, was 3.95, 3.97, 3.99, and 4.00 eV, for ZnNb_2_O_6_-9D, 7D, 5D, and 3D, respectively. One possible explanation to the slight increase in bandgap energy from ZnNb_2_O_6_-9D to ZnNb_2_O_6_-3D is the quantum effect, as can be seen in the SEM images.

**Fig. 4:**
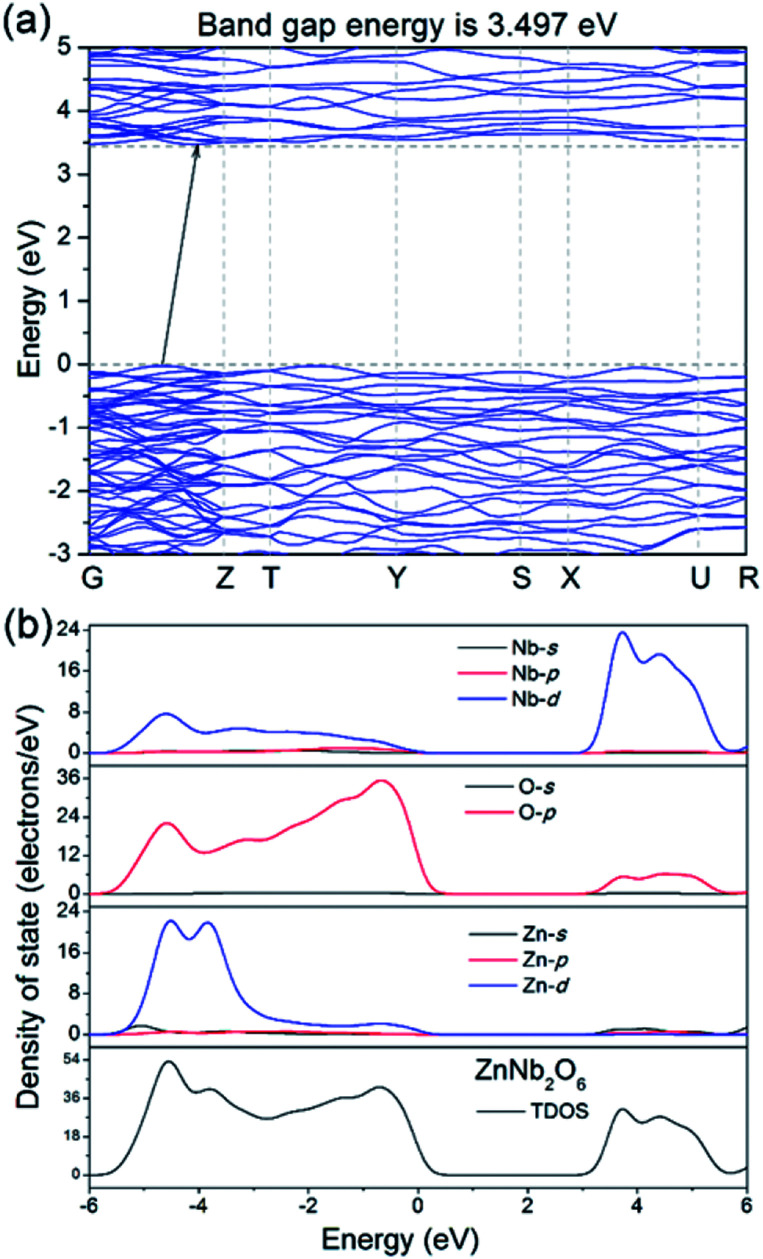
(a) Calculated energy band structure of ZnNb_2_O_6_, (b) partial and total density of state in the range from −6 to 6 eV.

DFT calculations help the understanding of the band structure and the possible electronic conduction mechanism. As shown in Fig. 4a, it is an indirect semiconductor with the calculated bandgap energy of 3.497 eV, smaller than the estimated ones by DRS spectra, which is commonly seen due to the discontinuity of the XC energy during the calculations. In addition, the calculated partial density states (PDOS) in Fig. 4b indicates that the top of the valence band (VB) is dominated by O 2p orbitals, while the bottom of the conduction band (CB) is mainly dominated by the Nb 5d orbitals. This is understandable and it suggests that the electronic conduction is mainly dominated by the Nb–O covalent bonds, *i.e.* photo-excited electrons/holes probably move along the NbO_6_ octahedra-based zig–zag chains.

**Fig. 5:**
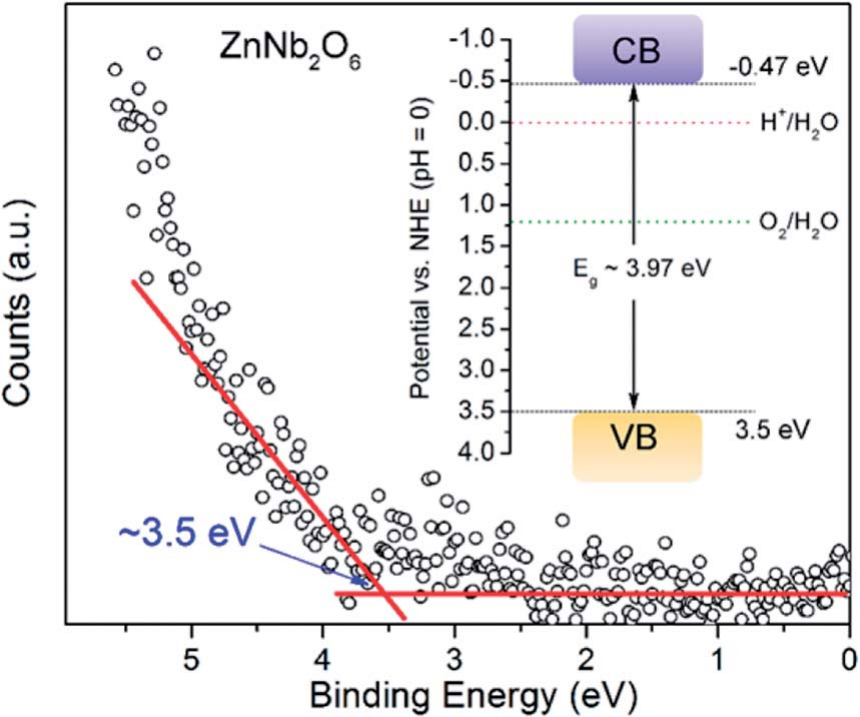
Potential of valence electron for ZnNb_2_O_6_ was estimated according to the rising point in ultraviolet photoelectron spectrum, and the proposed band structure potentials are provided as the inset.

Apparently, both potentials for VB and CB of ZnNb_2_O_6_ are sufficient for the water oxidation and reduction, respectively, since the water splitting activity has been reported in literature.^9^ Here, ultraviolet photoelectron spectroscopy (UPS) was applied to acquire the actual potential of the valence electron. We propose that the VB potential is roughly 3.5 eV according to Fig. 5. In combination with the experimental value of bandgap energy from DRS (*i.e.* 3.97 eV), the CB potential is −0.47 eV (see the inset of Fig. 5).

In order to further corroborate the correctness of these values, a semi-empirical calculation based on Mulliken electronegativity^21^ was performed using the following equations:

1 *E*_VB_ = *χ* − *E*_e_ + 1/2*E*_g_

2 *E*_CB_ = *E*_VB_ − *E*_g_

*E*_VB_ and *E*_CB_ are potentials of VB and CB edges, respectively. *χ* is Mulliken electronegativity, which is 6.18 eV according to its formula. *E*_e_ is the energy of free electrons on the hydrogen scale (4.5 eV). *E*_g_ is band gap value obtained from the DRS (*i.e.* 3.97 eV). Accordingly, the as-estimated *E*_VB_ and *E*_CB_ are 3.665 eV and −0.305 eV, which are generally consistent with the obtained values from the UPS method.

### Photocatalytic performances of as-synthesized ZnNb_2_O_6_

**Fig. 6:**
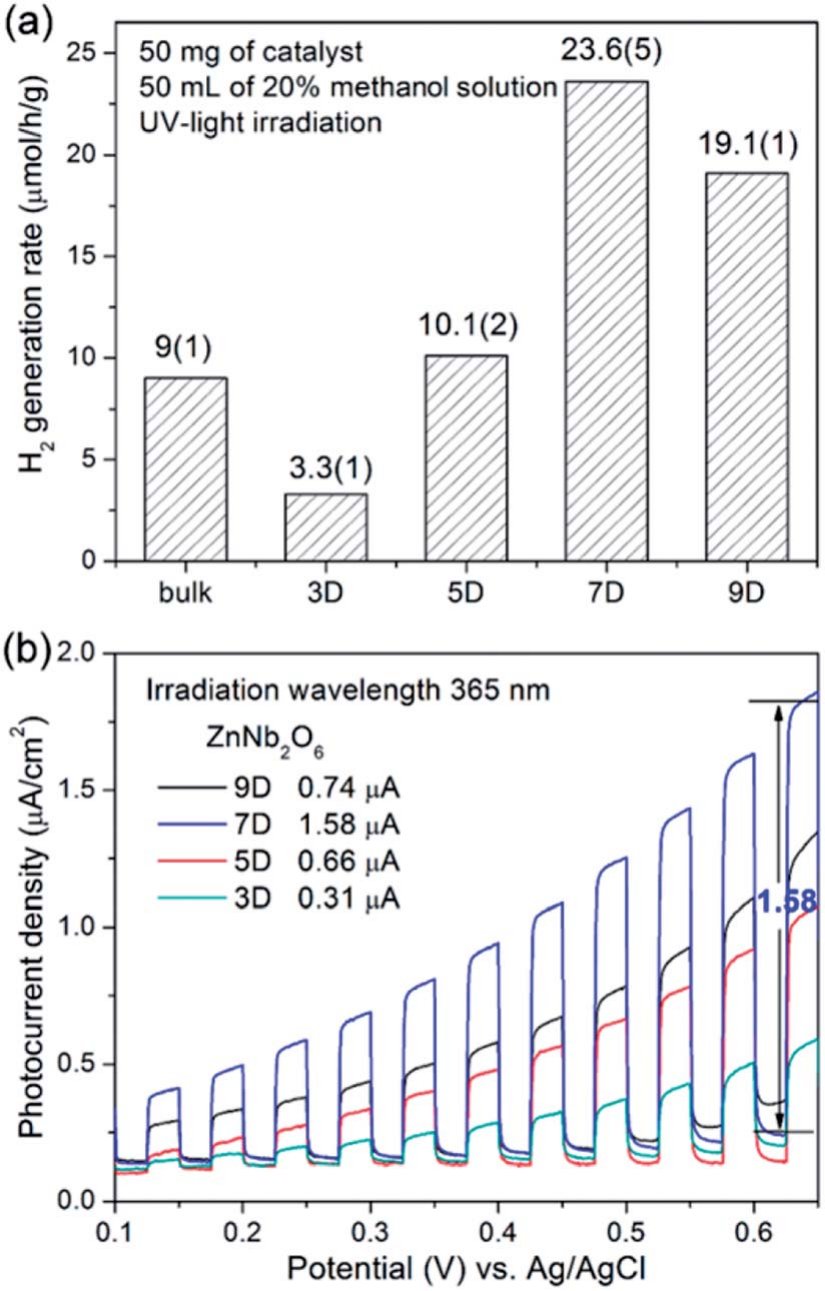
(a) Photocatalytic H_2_ generation rates of bulk-ZnNb_2_O_6_ and nano-ZnNb_2_O_6_ synthesized hydrothermally for 3, 5, 7, and 9 days, (b) photoelectrochemical responses using a three-electrode setup in 0.1 M Na_2_SO_4_ aqueous solution.

Under UV irradiation, all four as-synthesized samples (ZnNb_2_O_6_-3D, -5D, -7D, and -9D) as well as the bulk-type ZnNb_2_O_6_ obtained from high temperature solid state reaction were active to catalyze the H_2_ generation with the assistance of methanol (20 vol%) as the sacrificial agent. As shown in Fig. 6a, the hydrothermal samples exhibited the optimal activity of 23.6(5) μmol h^−1^ g^−1^, while the H_2_ generation rate of the bulk ZnNb_2_O_6_ was 9(1) μmol h^−1^ g^−1^. Time-dependent H_2_ evolution data are provided in Fig. S4, ESI.† First, the performance of nano-type ZnNb_2_O_6_ was in the same level with the bulk-type sample, although we claimed the great superiority of nanomaterials for photocatalysis. Indeed, the nano-type samples all have very large BET surfaces, however, the probably insufficient crystal growth due to the relatively low reaction temperature will lead to a large number of surficial defects, *i.e.* the ionic vacancies on the surface, which could be the electron–hole recombination centers. In synthetic chemistry, we could extend the reaction time to overcome this drawback partially. According to SEM and BET experiments, the increase of the crystallinity will decrease the specific surface area. Eventually, ZnNb_2_O_6_-7D possesses the highest performance under current conditions. This claim could be also proved by the photocurrent measurements in the external electric field, which is a well-known method to understand the generation, separation, and migration of photo-excited charges of semiconductors.^22,23^ As shown in Fig. 6b, the photocurrents of as-prepared ZnNb_2_O_6_ were recorded in dark with a set of chopped linear-sweeps with a scan rate of 10 mV s^−1^ between 0.0–0.65 V in 0.1 M Na_2_SO_4_ solution. The photocurrent responses also exhibited a maximum value for the sample ZnNb_2_O_6_-7D. Accordingly, it was selected as the best sample in quality for the following experiments.

### Cocatalysts loading on ZnNb_2_O_6_-7D

As mentioned above, the major problem of the nano-ZnNb_2_O_6_ is the high level of surficial defects, which first could be partially relieved by extending the reaction time. On the other hand, the most commonly used strategy is to post-modify the catalyst powder with specific cocatalysts, *i.e.* metal or metal oxides. It is well known that appropriate loading of co-catalyst on the photocatalysts not only facilitates the surficial charge separation but also offering a large number of catalytic sites, both are positive effects to photocatalysis.^24^

**Fig. 7:**
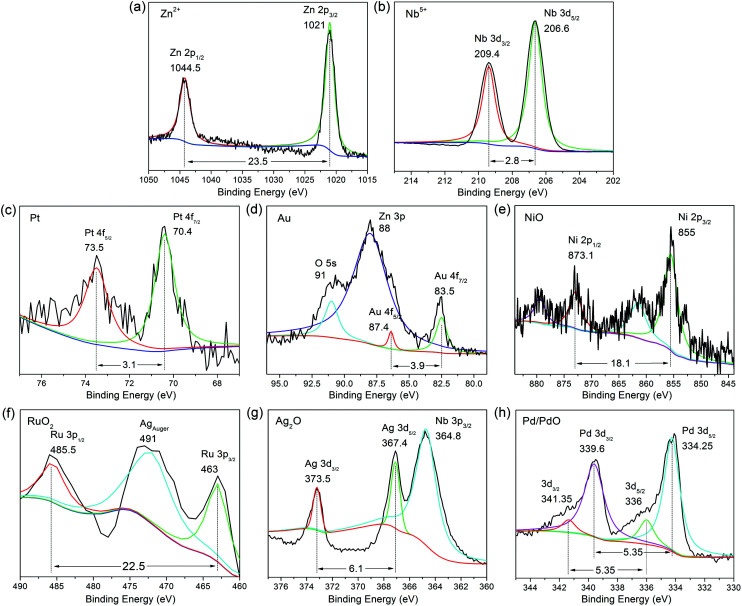
XPS spectra for (a and b) ZnNb_2_O_6_ loaded with (c) Pt, (d) Au, (e) NiO, (f) RuO_2_, (g) Ag_2_O, and (h) Pd/PdO.

Six commonly used elements were selected (including Pt, Au, Ru, Ni, Pd, Ag)^25–30^ to be deposited on the surface of ZnNb_2_O_6_-7D in quantity of 1 wt%. The successful loading and the valence states of these elements were investigated by X-ray photoelectron spectroscopy, which is a useful surface-detecting technique. As expected, Zn^2+^ and Nb^5+^ are in their usual valence states as shown in Fig. 7a and b. For instance, Zn 2p level split into two peaks of Zn 2p_3/2_ and Zn 2p_1/2_ due to spin-orbital coupling, which are positioned at 1021 and 1044.5 eV, respectively. It is the same with Nb 3d level, *i.e.* Nb 3d_5/2_ and Nb 3d_3/2_ locate at 206.6 and 209.4 eV respectively. The signals for the loaded elements are detectable but very weak. As shown in Fig. 7c–h, the cocatalysts are supposed to be Pt, Au, NiO, RuO_2_, Ag_2_O, respectively. The case of Pd is somehow different, where the coexistence of Pd and PdO is proposed on the basis of its peak broadening of the binding energy.

**Fig. 8:**
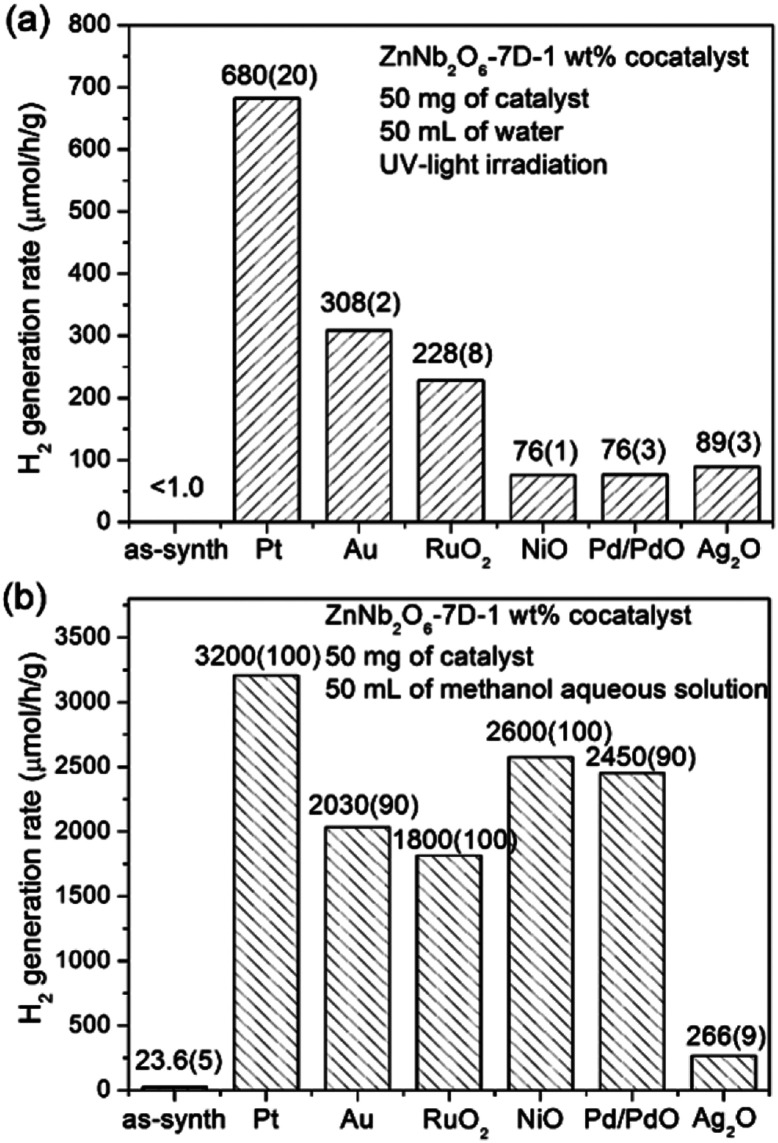
Photocatalytic H_2_ evolution rates of ZnNb_2_O_6_-7D loaded with 1 wt% co-catalyst in (a) pure water, (b) 20 vol% methanol solution under UV light irradiation.

The photocatalytic H_2_ generation performances of ZnNb_2_O_6_-7D loaded with 1 wt% cocatalysts either in pure water or in 20 vol% methanol aqueous solution are presented in Fig. 8. Detailed time-dependent H_2_ evolution data are provided in Fig. S5, ESI.† First, the photocatalytic activity of as-prepared ZnNb_2_O_6_-7D was almost undetectable (<1 μmol h^−1^ g^−1^) in pure water, while it increased to 23.6(5) μmol h^−1^ g^−1^ when using methanol as the sacrificial agent. This increase is due to the fast consuming of surficial holes by methanol, which, in some content, decreases the recombination rate of surficial charges. Second, the employment of cocatalysts is extremely helpful to enhance the photocatalytic H_2_ generation efficiency. The H_2_ generation rate achieved up to 680(20) and 3200(100) μmol h^−1^ g^−1^ in pure water or methanol aqueous solution when loading with 1 wt% Pt. Additional experiments were performed to find the optimal loading content of Pt. The colour of Pt-loaded samples turned from white to light grey, and finally to dark grey when increasing the weight percentage of Pt up to 2.0 wt%. The sample with 1 wt% Pt exhibited the optimal photocatalytic activity both in pure water and in methanol aqueous solution (see Fig. S6, ESI†). It is proposed that the over-abundant co-catalyst would result in large and aggregated Pt particles, thus the light absorbance and the number of effective catalytic sites would decrease. Here in our experiments, the optimal H_2_ generate rate can be achieved when the loading content of Pt is 1 wt%.

### Apparent quantum yields

Our intention is to explore the potential of ZnNb_2_O_6_ in photocatalytic water splitting when synthesized as nanomaterials. It is agreed that the photocatalytic H_2_ generation efficiency could be affected by the experimental conditions, thus people prefer to use a semi-quantitative parameter, the apparent quantum yield (AQY), to evaluate the photocatalytic efficiency of a specific catalyst. It is a semi-quantitative parameter because people cannot obtain an accurate number of absorbed photons. Practically, we need to adjust the experimental conditions to get an optimal AQY value, such as the quantity of the photocatalyst and aqueous solution, and the beam intensity of the irradiation light. Under the monochromatic incident beam of 295 nm, the H_2_ evolution rates as well as the AQYs are provided in Table 1, where the dosage of the catalyst (ZnNb_2_O_6_-7D loaded with 1 wt% Pt) as well as the volume of water are variable. A maximal AQY value of 4.54% was obtained when using 110 mg of catalyst and in 100 mL of pure water. When varying the incident beam intensity, the AQY of 4.54% remained the optimal in pure water (see Table 2). In the following, we obtained the highest AQY of 9.25% with the assistance of methanol as the sacrificial agent (see Table 3 for detailed experimental condition).

**Table 1:** Calculated AQYs of ZnNb_2_O_6_-7D-1 wt% Pt in pure water when varying the dose of catalyst and the volume of water

Light intensity (mW cm^−2^)	Catalyst (mg)	Water (mL)	H_2_ generation rate (μmol h^−1^)	AQY (%)
2.298	50	50	2.4(2)	1.18
	60	50	1.9(1)	0.93
	60	60	2.6(3)	1.29
	70	60	3.4(3)	1.67
	70	70	3.3(3)	1.65
	80	70	4.3(4)	2.14
	80	80	5.6(4)	2.79
	90	80	4.8(4)	2.38
	90	90	3.9(3)	1.95
	100	90	6.2(8)	3.07
	100	100	5.5(5)	2.76
	110	100	9.1(6)	4.54
	110	110	7.8(5)	3.91
	120	110	9(1)	4.41
	140	110	5.4(7)	2.72

**Table 2:** Calculated AQYs of ZnNb_2_O_6_-7D-1 wt% Pt in pure water by changing the incident light density

Light intensity (mW cm^−2^)	Catalyst (mg)	Water (mL)	H_2_ generation rate (μmol h^−1^)	AQY (%)
1.335	110	100	3.9(5)	3.35
2.298	110	100	9.1(6)	4.54
3.353	110	100	7.2(9)	2.47

**Table 3:** Calculated AQYs of ZnNb_2_O_6_-7D-1 wt% Pt in 20 vol% methanol aqueous solution by changing the incident light intensity

Light intensity (mW cm^−2^)	Catalyst (mg)	20 vol% methanol aq. (mL)	H_2_ generation rate (μmol h^−1^)	AQY (%)
0.659	110	100	4.5(3)	7.80
1.998	110	100	16(2)	9.25
4.319	110	100	28(3)	7.45

**Fig. 9:**
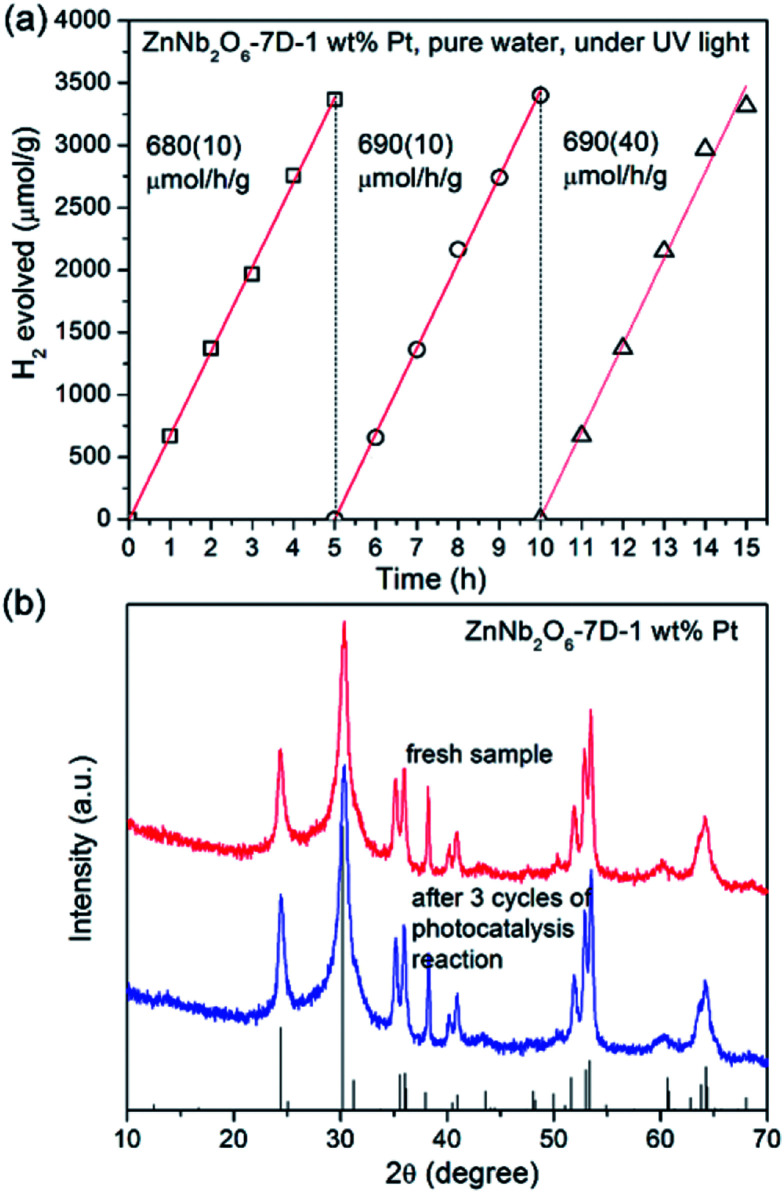
(a) Time-dependent H_2_ evolution curves for ZnNb_2_O_6_-7D-1 wt% Pt in pure water. After each 5 hours, the photocatalysis system was evacuated. (b) XRD patterns for fresh ZnNb_2_O_6_-7D-1 wt% Pt and the recovered sample after 15 hours photocatalysis experiment.

Finally, the stability of photocatalytic activity and the integrity of the sample crystallinity is very important for a heterogeneous catalyst. As shown in Fig. 9, the photocatalytic activity of ZnNb_2_O_6_-7D loaded with 1 wt% Pt remained constant for at least three cycles of photocatalytic experiments (15 hours of irradiation). Moreover, the XRD collected for the recovered sample exhibited an identical pattern with the fresh sample, indicating that there was no photo-corrosion under our experimental condition.

## Conclusions

The two-step hydrothermal method was employed to synthesize flower-like nanosized ZnNb_2_O_6_ in order to enhance its photocatalytic performance in water reduction. Experimentally, ZnNb_2_O_6_-7D exhibited the optimal photocatalytic performance among the samples synthesized hydrothermally with different reaction times, due to its appropriate crystallinity and high specific surface area, for example, the photocatalytic H_2_ generation rate was 23.6(5) μmol h^−1^ g^−1^, which is indeed larger than that of bulk-ZnNb_2_O_6_ under the same catalytic condition. However, the drawback of the nano-ZnNb_2_O_6_ is also very obvious, that is the severe surficial defects due to the low preparation temperature. Consequently, various cocatalysts, including Pt, Au, NiO, RuO_2_, Ag_2_O, Pd/PdO, were deposited on the catalyst surface by reduction method using KBH_4_ solution. It is impressive that the H_2_ generation rate increased for two orders, that is 680(20) and 3200(100) μmol h^−1^ g^−1^ in pure water and in methanol aqueous solution, respectively. A systematic investigation of AQYs for ZnNb_2_O_6_-7D-1 wt% Pt was performed by varying the experimental conditions, and the optimal AQYs were 4.54% and 9.25% in water and in methanol solution, respectively.

## Conflicts of interest

There are no conflicts to declare.

## Supplementary Material

RA-008-C8RA01624K-s001
