# Supplementary material for: Optimizing the performance of photocatalytic H2 generation for ZnNb2O6 synthesized by a two-step hydrothermal method
Source: RSC Adv. 2018 Apr 13;8(25):13857–64. doi: 10.1039/c8ra01624k (PMC9079817; doi:10.1039/c8ra01624k)
Supplement: RA-008-C8RA01624K-s001 [file RA-008-C8RA01624K-s001.pdf]

Electronic Supplementary Material (ESI) for RSC Advances.
This journal is © The Royal Society of Chemistry 2018

Electronic Supplementary Information for

Optimizing the performance of photocatalytic H_2 generation for $ZnNb_2O_6$ synthesized by a two-step hydrothermal method

Yutong Chun,[a] *Mufei Yue*[a] *Pengfei Jiang,*[ab*] *Shijian Chen,*[b] *Wenliang Gao,*[a] *Rihong Cong,*[a] *Tao Yang*[a*]

[a] College of Chemistry and Chemical Engineering, Chongqing University, Chongqing 401331, P. R. China

[b] College of Physics, Chongqing University, Chongqing 401331, P. R. China

[*]Corresponding authors, e-mails: pengfeijiang@cqu.edu.cn; taoyang@cqu.edu.cn.

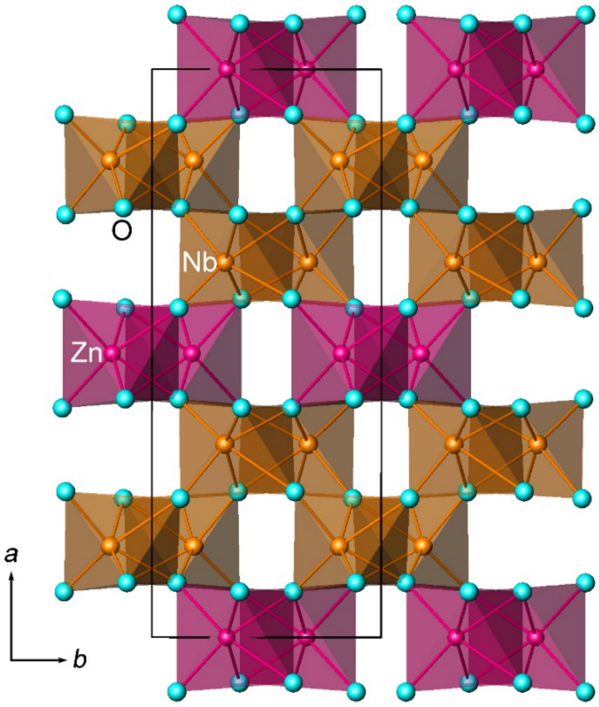

Figure S1 Structure view of $ZnNb_2O_6$ along the *c*-axis.

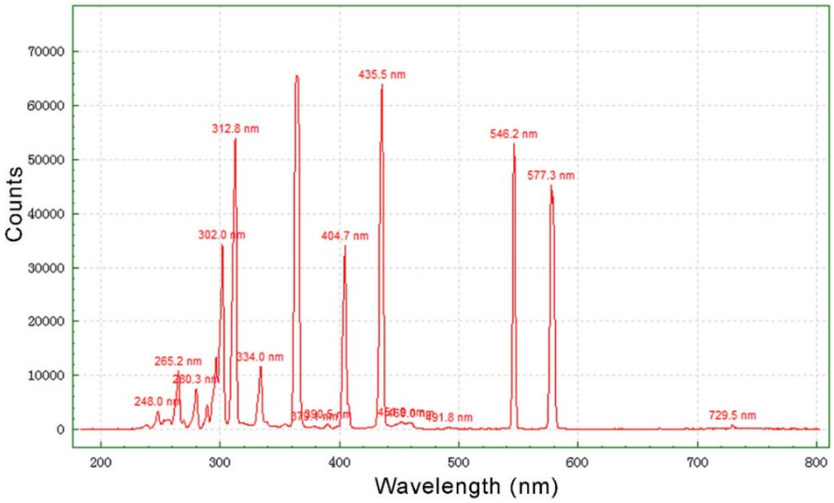

Figure S2 Emission spectrum from the Hg-lamp used in our study.

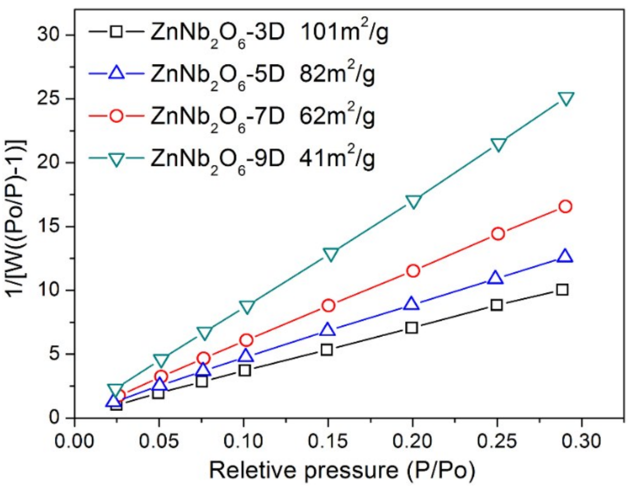

Figure S3 Specific surface areas for $ZnNb_2O_6$-3D, -5D, -7D, and -9D estimated according to the BET method.

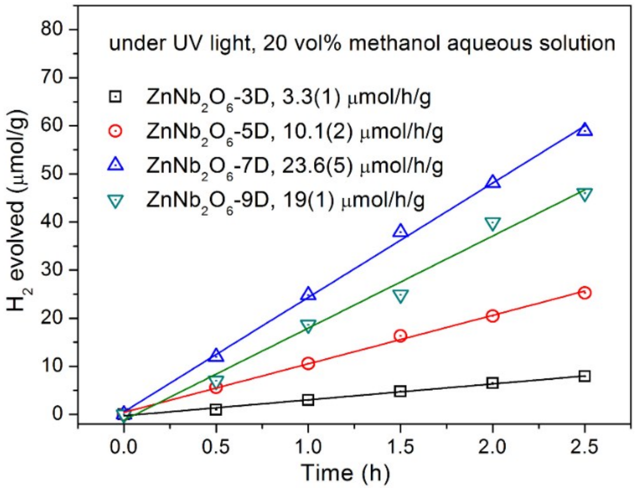

Figure S4 Time-dependent photocatalytic H_2 generation data for as-synthesized $ZnNb_2O_6$ samples.

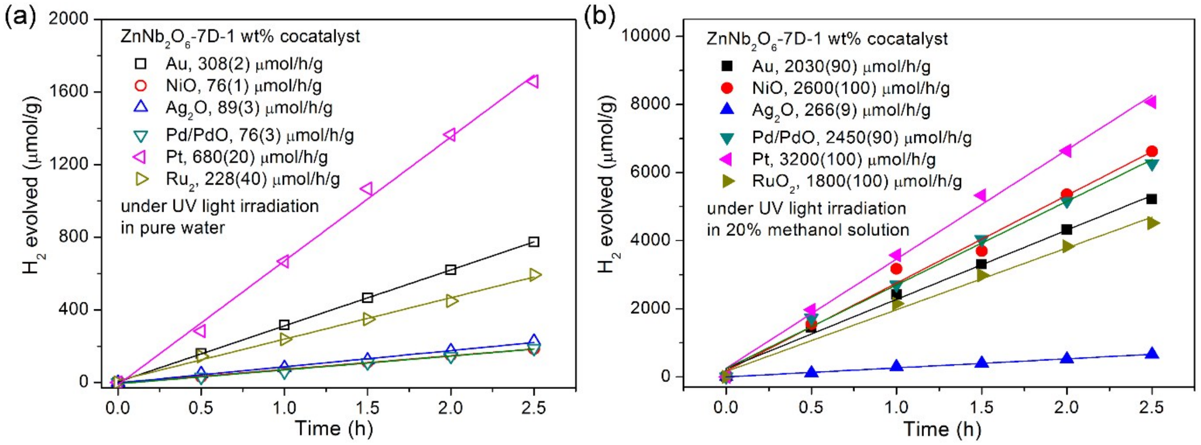

Figure S5 Time-dependent photocatalytic H_2 generation data for $ZnNb_2O_6$-loaded with 1 wt% cocatalyst in (a) water and (b) 20 vol% methanol aqueous solution.

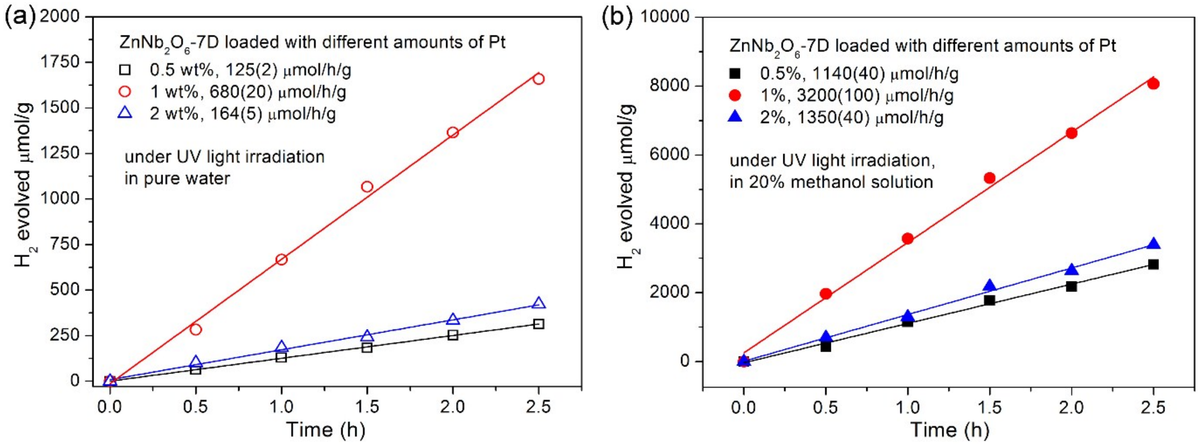

Figure S6 Photocatalytic H_2 evolution curves of $ZnNb_2O_6$-7D loaded with different amounts of Pt in (a) water and (b) 20 vol% methanol solution.
